# Supplementary material for: Time-Dependent Analysis of Plasmalogens in the Hippocampus of an Alzheimer’s Disease Mouse Model: A Role of Ethanolamine Plasmalogen
Source: Brain Sci. 2021 Dec 2;11(12):1603. doi: 10.3390/brainsci11121603 (PMC8699479; doi:10.3390/brainsci11121603)
Supplement: Supplementary file 1 [file brainsci-11-01603-s001.zip › supplementary materials.pdf]

Supplemental Figure

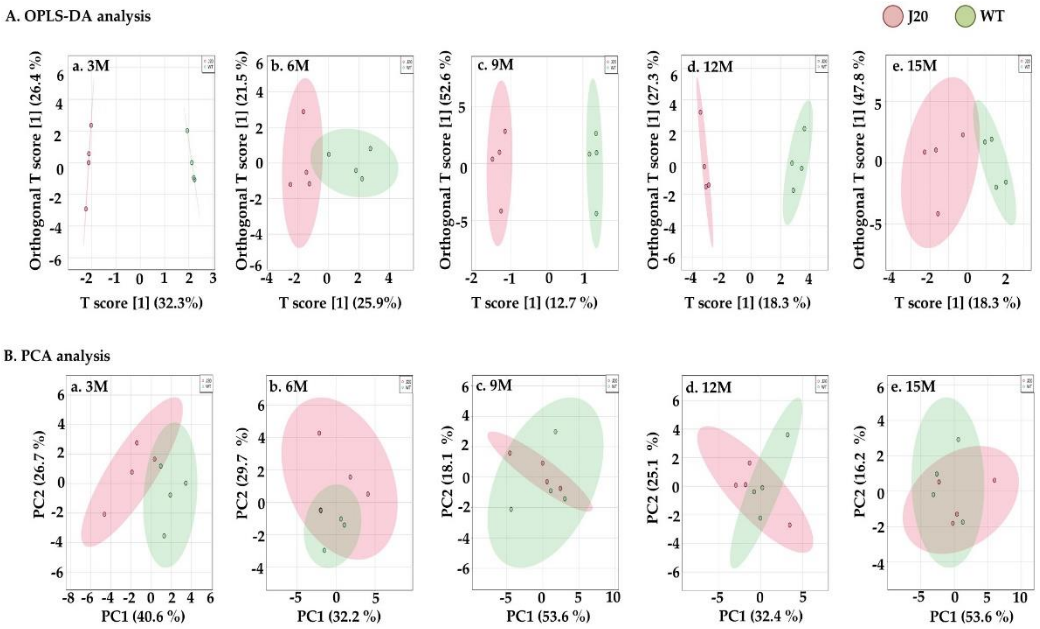

Figure S1: Orthogonal partial least square discriminant analysis (OPLS-DA) and principal component analysis (PCA) of Pls-PC in the J20 and WT mice**.** Four mice were used in each group for J20 and WT mice at each time point. OPLS-DA analysis (**A)** at 3M (A.a), 6M (A.b), 9M (A.c), 12M (A.d), 15M (A.e), and PCA analysis (**B)** at 3M (B.a), 6M (B.b), 9M (B.c), 12M (B.d), 15M (B.e) were conducted with the measured Pls-PC by using MetaboAnalyst 5.0 software. Pink-colored circles indicate Pls-PC distribution in J20 mice, and green-colored circles indicate Pls-PC distribution in WT mice. M indicates months.

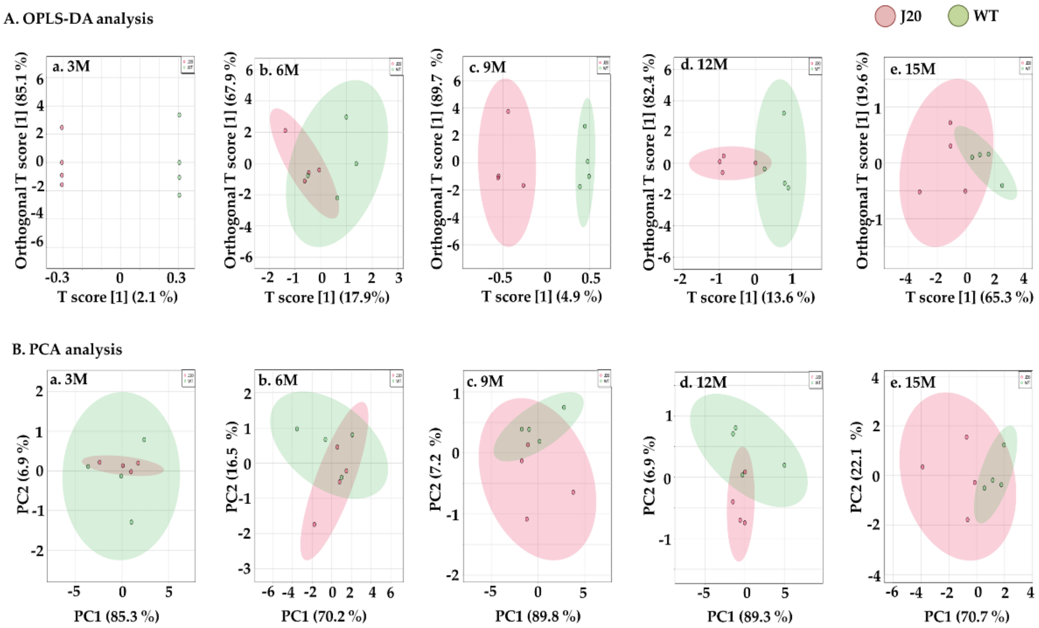

Figure S2: Orthogonal partial least square discriminant analysis (OPLS-DA) and principal component analysis (PCA) of the alkyl-acyl-PC in the J20 and WT mice. Four mice were used in each group for J20 and WT mice at each time point. OPLS-DA analysis (**A**) at 3M (A.a), 6M (A.b), 9M (A.c), 12M (A.d),

15M (A.e), and PCA analysis (**B)** at 3M (B.a), 6M (B.b), 9M (B.c), 12M (B.d), 15M (B.e) were conducted with the measured alkyl-acyl-PC by using MetaboAnalyst 5.0 software. Pink-colored circles indicate alkyl-acyl-PC distribution in J20 mice, and green-colored circles indicate alkyl-acyl-PC distribution in WT mice. M indicates months.

Supplemental Table

Table S1. Pls-PE and alkyl-acyl-PE molecular species concentration (pmol/mg (±SD) in the hippocampus of wild-type and J20 mice.

Lipid Name	m/z	Molecular Species	3M		6M		9M		12M		15M	
			WT. (±SD)	J20 (±SD)	WT (±SD)	J20 (±SD)	WT (±SD)	J20 (±SD)	WT (±SD)	J20 (±SD)	WT (±SD)	J20 (±SD)
PE (P-34:1)	701	P-18:0/16:1	1.22 (0.32)	0.95 (0.38)	1.02 (0.32)	0.79 (0.27)	0.77 (0.26)	1.18 (0.69)	0.78 (0.41)	0.87 (0.22)	1.55 (0.35)	0.84[b] (0.17)
		P-16:0/18:1	13.32 (3.46)	14.01 (5.61)	11.37 (3.62)	9.94 (3.41)	9.46 (3.21)	15.12 (8.87)	10.10 (5.25)	10.18 (2.59)	18.29 (4.10)	10.70[b] (2.19)
PE (P-34:0)	699	P-18:0/16:0	23.27 (9.23)	25.64 (6.71)	20.42 (3.64)	21.36 (3.98)	17.23 (5.97)	27.83 (12.24)	22.95 (6.47)	20.57 (5.73)	30.65 (8.39)	20.15[a] (4.30)
		P-16:0/18:0	1.34 (0.53)	2.63[a] (0.69)	1.05 (0.19)	1.58[a] (0.29)	1.69 (0.59)	2.16 (0.95)	2.10 (0.59)	1.80 (0.50)	2.75 (0.75)	1.95 (0.42)
PE (P-36:4)	723	P-16:0/20:4	13.66 (1.35)	15.85 (2.14)	14.82 (4.56)	18.16 (5.31)	13.00 (1.40)	17.24[b] (2.15)	11.22 (2.01)	15.65 (3.71)	15.67 (4.23)	12.79 (4.62)
PE (P-36:3)	725	P-16:0/20:3	9.96 (2.18)	13.67 (6.87)	12.98 (5.61)	11.97 (2.47)	9.28 (1.08)	12.98[b] (1.86)	9.70 (1.60)	9.97 (2.92)	12.78 (4.51)	8.46 (2.90)
		P-18:1/18:2	3.72 (0.81)	3.14 (1.58)	5.84 (2.52)	6.78 (1.40)	4.52 (0.53)	8.38[b] (1.20)	4.93 (0.81)	4.26 (1.25)	4.49 (1.58)	3.90 (1.34)
PE (P-36:2)	727	P-16:0/20:2	0.50 (0.08)	0.69[a] (0.12)	0.58 (0.11)	0.42[a] (0.04)	0.28 (0.08)	0.53[a] (0.21)	0.45 (0.06)	0.40 (0.06)	0.59 (0.10)	0.45[a] (0.04)
		P-18:0/18:2	0.67 (0.10)	0.83 (0.14)	0.95 (0.18)	0.61[b] (0.05)	0.45 (0.12)	0.79[a] (0.31)	0.57 (0.08)	0.45[a] (0.07)	0.58 (0.10)	0.42[b] (0.04)
		P-18:1/18:1	45.12 (6.97)	45.71 (7.96)	66.36 (12.50)	47.92[a] (4.24)	53.89 (14.41)	80.60 (31.78)	74.56 (10.6)	56.27[a] (8.41)	97.59 (16.62)	57.84[b] (4.92)
PE (P-36:1)	729	P-16:0/20:1	5.35 (2.16)	7.19 (1.99)	3.48 (0.38)	3.88 (0.54)	3.91 (1.21)	7.85 (6.28)	5.94 (3.21)	4.15 (1.00)	8.17 (2.22)	4.85[a] (1.66)
		P-18:0/18:1	36.65 (14.78)	41.67 (11.5)	29.79 (3.27)	26.52 (3.72)	23.76 (7.36)	45.04 (36.04)	34.61 (18.73)	22.45 (5.38)	46.37 (12.58)	26.99[a] (9.26)
		P-18:1/18:0	1.72 (0.69)	1.75 (0.49)	1.60 (0.18)	1.75 (0.25)	1.49 (0.46)	2.81 (2.25)	2.66 (1.44)	1.78 (0.43)	3.75 (1.02)	2.10[a] (0.72)
PE (P-36:0)	731	P-16:0/20:0	1.31 (0.38)	3.23[b] (0.82)	2.15 (0.39)	2.74 (0.59)	2.01 (0.49)	2.65 (1.54)	3.18 (1.46)	2.73 (0.38)	3.59 (0.41)	3.39 (0.38)
		P-18:0/18:0	13.61 (3.94)	18.65 (4.74)	17.54 (3.18)	16.55 (3.57)	13.37 (3.26)	26.66 (15.52)	20.24 (9.29)	15.18 (2.12)	21.70 (2.48)	16.20[b] (1.83)
PE (P-38:6)	747	P-16:0/22:6	36.53 (21.38)	36.08 (14.76)	26.64 (4.75)	21.62 (3.10)	44.92 (18.30)	50.68 (23.84)	24.19 (2.53)	26.47 (6.77)	26.08 (4.65)	21.63 (4.09)
		P-18:1/20:5	0.25 (0.15)	0.22 (0.09)	0.19 (0.03)	0.19 (0.03)	0.50 (0.20)	0.40 (0.19)	0.18 (0.02)	0.20 (0.05)	0.18 (0.03)	0.13[b] (0.02)
PE (P-38:5)	749	P-16:0/22:5	26.68 (10.79)	34.49 (17.26)	39.88 (2.93)	35.42 (3.95)	22.17 (3.97)	31.36[a] (6.33)	34.11 (10.56)	34.12 (12.62)	34.62 (17.73)	31.14 (11.40)
		P-18:0/20:5	2.40 (0.97	2.76 (1.38)	3.79 (0.28)	2.68[b] (0.30)	1.54 (0.29)	2.34[a] (0.47)	2.15 (0.66)	1.91 (0.71)	1.97 (1.01)	2.01 (0.73)
		P-18:1/20:4	129.09 (52.20)	152.89 (76.49)	250.24 (18.37)	249.85 (27.86)	194.42 (36.50)	251.16 (50.65)	197.19 (61.05)	191.76 (70.90)	218.70 (112.02)	187.42 (68.59)
PE (P-38:4)	751	P-16:0/22:4	20.64 (4.07)	24.57 (4.12)	27.94 (3.26)	27.29 (4.77)	17.19 (1.08)	25.56[b] (2.35)	22.35 (2.05)	24.26 (5.66)	25.34 (3.87)	22.65 (4.72)
		P-18:0/20:4	70.79 (13.96)	65.26 (10.94)	87.35 (10.18)	83.72 (14.65)	63.62 (3.99)	84.04[b] (7.74)	63.98 (5.86)	56.57 (13.20)	68.50 (10.46)	58.71 (12.23)
		P-18:1/20:3	4.52 (0.89)	5.59 (0.94)	6.66 (0.78)	7.65 (1.34)	7.90 (0.50)	8.41 (0.78)	7.52 (0.69)	7.16 (1.67)	9.69 (1.48)	8.51 (1.77)
PE (P-38:3)	753	P-16:0/22:3	5.85 (1.04)	6.13 (0.96)	9.17 (0.53)	10.07 (1.21)	5.63 (0.66)	8.34[b] (1.56)	6.72 (0.66)	7.56 (1.32)	9.15 (0.38)	7.58[b] (0.88)
		P-18:0/20:3	42.61 (7.56)	47.74 (7.45)	50.25 (2.89)	50.64 (6.08)	33.52 (3.91)	44.99[a] (8.42)	33.24 (3.26)	31.73 (5.54)	37.96 (1.58)	32.00[a] (3.73)
		P-18:1/20:2	25.76	29.90	33.78	38.20	27.57	39.7[a]	31.82	32.67	35.89	28.00[b]

			(4.57)	(4.66)	(1.94)	(4.58)	(3.22)	(7.43)	(3.12)	(5.70)	(1.50)	(3.27)
PE (P-38:2)	755	P-18:0/20:2	1.16 (0.42)	1.87[a] (0.27)	1.87 (0.24)	1.20[b] (0.18)	1.27 (0.91)	1.39 (0.53)	1.30 (0.19)	1.05 (0.28)	5.08 (1.62)	2.72[a] (0.57)
		P-18:1/20:1	15.45 (5.55)	22.54[a] (3.26)	28.94 (3.76)	22.54[a] (3.47)	33.81 (24.23)	37.20 (14.18)	33.61 (4.91)	24.38[a] (6.56)	42.37 (13.51)	26.19[a] (5.52)
PE (P-40:7)	773	P-18:1/22:6	24.02 (3.34)	22.77 (3.94)	24.46 (1.66)	25.08 (2.59)	29.52 (6.75)	32.20 (9.30)	29.33 (2.10)	27.93 (2.05)	32.78 (4.47)	28.70 (3.51)
PE (P-40:6)	775	P-16:0/24:6	0.41 (0.18)	0.56 (0.15)	0.21 (0.05)	0.18 (0.04)	0.27 (0.05)	0.42[b] (0.08)	0.31 (0.07)	0.32 (0.05)	0.36 (0.08)	0.42 (0.06)
		P-18:0/22:6	167.74 (74.67)	175.76 (47.50)	106.54 (24.34)	93.39 (22.26)	171.97 (33.18)	215.74[a] (38.29)	156.80 (37.08)	161.02 (25.74)	182.01 (42.40)	168.55 (25.80)
		P-18:1/22:5	3.12 (1.39)	3.87 (1.05)	2.38 (0.54)	2.22 (0.53)	5.55 (1.07)	6.36 (1.13)	4.21 (1.00)	4.49 (0.72)	5.51 (1.28)	4.99 (0.76)
PE (P-40:5)	777	P-16:0/24:5	1.87 (0.11)	2.70[a] (0.61)	2.64 (0.26)	2.12[a] (0.21)	0.97 (0.09)	1.73[b] (0.09)	1.43 (0.11)	1.56 (0.22)	1.17 (0.30)	1.55 (0.25)
		P-18:0/22:5	58.18 (3.44)	53.79 (12.17)	71.75 (7.06)	61.18[a] (6.03)	39.67 (3.50)	52.05[b] (2.69)	45.80 (3.36)	38.88[a] (5.52)	46.44 (11.89)	39.67 (6.51)
		P-18:1/22:4	80.99 (4.79)	93.15 (21.07)	108.94 (10.73)	116.85 (11.51)	103.57 (9.14)	128.27[b] (6.63)	115.52 (8.48)	102.61 (14.57)	128.76 (32.96)	106.08 (17.41)
		P-20:1/20:4	2.91 (0.17)	2.74 (0.62)	3.95 (0.39)	4.07 (0.40)	3.14 (0.28)	3.72[a] (0.19)	3.68 (0.27)	2.58[b] (0.37)	3.62 (0.93)	3.30 (0.54)
PE (P-40:4)	779	P-16:0/24:4	0.73 (0.07)	0.92[a] (0.13)	0.68 (0.11)	0.79 (0.11)	0.44 (0.11)	0.59 (0.14)	0.67 (0.05)	0.60 (0.10)	0.78 (0.11)	0.47[b] (0.05)
		P-18:0/22:4	37.56 (3.82)	39.10 (5.69)	37.12 (5.85)	48.57[a] (6.93)	30.90 (7.79)	37.68 (8.61)	32.92 (2.25)	31.23 (5.10)	36.27 (5.17)	27.60[a] (2.74)
		P-18:1/22:3	1.84 (0.19)	2.29[a] (0.33)	2.98 (0.47)	4.91[b] (0.70)	5.10 (1.29)	5.32 (1.22)	4.80 (0.33)	4.64 (0.76)	6.46 (0.92)	5.14[a] (0.51)
		P-20:0/20:4	1.44 (0.15)	1.53 (0.22)	1.37 (0.22)	1.57 (0.22)	1.13 (0.28)	1.19 (0.27)	1.24 (0.08)	0.98[a] (0.16)	1.47 (0.21)	1.08[b] (0.11)
PE (P-40:3)	781	P-18:0/22:3	10.09 (1.68)	11.84 (2.83)	15.72 (0.92)	17.17 (2.85)	4.84 (0.90)	4.00 (0.42)	12.10 (1.91)	10.62 (3.77)	10.66 (2.52)	12.99 (4.35)
		P-18:1/22:2	15.32 (2.55)	17.63 (4.21)	23.68 (1.38)	26.85 (4.46)	24.24 (4.49)	29.72[a] (3.11)	16.98 (2.69)	18.17 (6.45)	15.06 (3.56)	17.52 (5.86)
Total Pls-PE			843.31 (102.31)	934.06 (79.67)	1025.75 (67.31)	1006.84 (79.11)	898.83 (125.78)	1203.48[a] (237.76)	959.55 (85.63)	877.55 (165.18)	1138.23 (83.90)	843.20[b] (47.61)
PE (O-34:0)		P-18:0/16:0	0.92 (0.23)	1.52[a] (0.38)	1.27 (0.42)	2.35 (1.18)	0.77 (0.44)	1.93[a] (0.67)	1.54 (0.53)	1.70 (0.92)	2.33 (1.42)	2.66 (0.92)

Note: Data presented here as Mean ± SD of 4 mice in a group. Statistical analysis was done with a paired *t*-test, where $p < 0.05$ was considered as significant. Statistical significance is denoted as follows: [a]$p < 0.05$, [b]$p < 0.01$. WT vs. J20.

Table S2. Pls-PC and alkyl-acyl-PC molecular species concentration (pmol/mg tissue (±SD)) in the hippocampus of wild-type and J20 mice.

Lipid Name	m/z	Molecular species	3M		6M		9M		12M		15M	
			WT (±SD)	J20 (±SD)	WT (±SD)	J20 (±SD)	WT (±SD)	J20 (±SD)	WT (±SD)	J20 (±SD)	WT (±SD)	J20 (±SD)
PC (P-32:1)	715	P-16:0/16:1	0.71 (0.12)	0.97 (0.12)	0.78 (0.10)	0.74 (0.09)	0.72 (0.19)	0.92 (0.23)	0.78 (0.03)	0.95 (0.14)	0.96 (0.17)	1.09 (0.27)
PC (P-32:0)	717	P-16:0/16:0	8.29 (0.87)	8.76 (0.84)	9.28 (0.99)	10.65 (1.40)	9.43 (0.53)	8.33[b] (0.38)	8.45 (0.75)	8.94 (0.47)	9.97 (1.35)	9.87 (1.34)
PC (P-34:1)	743	P-16:0/18:1	13.36 (1.31)	14.07 (1.86)	14.52 (1.00)	13.94 (2.48)	12.72 (3.49)	14.07 (2.15)	14.41 (0.80)	14.27 (1.50)	14.55 (1.70)	17.31 (3.21)
PC (P-34:0)	745	P-18:0/16:0	88.92 (7.43)	98.07 (3.24)	101.97 (3.86)	106.75 (11.02)	91.90 (12.08)	97.78 (9.05)	95.28 (8.31)	102.01 (10.93)	99.21 (15.01)	103.92 (10.22)
PC (P-36:5)	763	P-16:0/20:5	33.06 (1.33)	30.77 (2.51)	36.49 (4.98)	36.25 (3.47)	34.81 (2.75)	31.65 (3.88)	30.49 (2.82)	32.31 (3.36)	37.51 (4.89)	37.00 (1.99)
PC (P-36:4)	765	P-16:0/20:4	1.11 (0.17)	2.08[b] (0.20)	1.44 (0.13)	1.18[b] (0.08)	1.70 (0.54)	1.67 (0.47)	1.58 (0.34)	1.32 (0.16)	1.73 (0.31)	1.69 (0.78)
PC (P-36:3)	767	P-16:0/20:3	7.18 (0.72)	8.01 (0.73)	7.27 (1.39)	6.89 (1.01)	7.49 (1.12)	8.13 (0.71)	6.37 (1.49)	7.30 (0.81)	7.52 (0.80)	9.33[a] (0.83)

PC (P-36:2)	769	P-18:0/18:2	3.64 (0.85)	3.21 (0.60)	6.20 (0.75)	5.02^{a} (0.73)	5.91 (2.07)	6.68 (2.94)	7.21 (0.93)	6.37 (0.96)	7.10 (1.31)	7.79 (2.47)
PC (P-36:1)	771	P-18:0/18:1	5.17 (1.10)	5.42 (1.06)	7.43 (1.20)	5.87^{a} (0.95)	5.92 (1.76)	5.68 (1.39)	6.33 (0.57)	5.59 (0.95)	6.92 (1.30)	6.40 (1.71)
PC (P-36:0)	773	P-20:0/16:0	3.46 (0.60)	4.11 (1.55)	3.35 (0.64)	3.43 (0.91)	3.67 (1.87)	2.94 (0.59)	2.80 (0.41)	3.13 (1.05)	3.90 (1.66)	3.92 (1.01)
PC (P-38:6)	789	P-16:0/22:6	2.40 (0.19)	3.15^{b} (0.08)	3.88 (0.22)	4.70 (0.94)	4.34 (0.76)	3.38 (0.32)	4.17 (0.84)	3.98 (0.40)	3.96 (0.38)	4.53 (0.52)
PC (P-38:5)	791	P-16:0/22:5	12.63 (1.88)	14.42 (0.99)	15.67 (1.58)	15.32 (3.52)	15.69 (2.79)	16.91 (2.41)	16.13 (1.46)	16.47 (2.06)	17.95 (2.27)	20.68 (2.86)
PC (P-38:4)	793	P-18:0/20:4	0.67 (0.31)	0.58 (0.16)	1.79 (0.27)	1.42^{a} (0.14)	0.61 (0.38)	0.43 (0.10)	0.31 (0.24)	0.51 (0.12)	0.53 (0.11)	0.52 (0.17)
PC (P-38:3)	795	P-18:0/20:3	0.53 (0.13)	0.86 ^{a} (0.08)	1.64 (0.35)	1.68 (0.26)	0.85 (0.30)	0.95 (0.27)	1.03 (0.44)	1.15 (0.37)	1.26 (0.20)	1.51 (0.41)
PC (P-38:2)	797	P-18:0/20:2	0.40 (0.03)	0.43 (0.25)	1.36 (0.35)	0.88^{a} (0.32)	1.01 (0.43)	1.19 (0.32)	1.48 (0.12)	1.03^{b} (0.25)	1.79 (0.56)	1.69 (0.87)
PC (P-40:6)	817	P-18:0/22:6	0.46 (0.09)	0.39 (0.06)	0.56 (0.03)	0.62 (0.20)	0.72 (0.07)	0.78 (0.34)	0.71 (0.21)	0.63 (0.13)	0.65 (0.10)	0.82 (0.12)
Total Pls-PC			174.51 (12.51)	186.47 (5.30)	201.63 (12.41)	204.66 (22.23)	187.14 (22.76)	191.63 (20.70)	186.75 (11.77)	195.88 (18.82)	203.60 (24.08)	215.64 (17.39)
PC (O-32:0)	719	O-16:0/16:0	75.06 (9.41)	72.90 (11.86)	76.87 (6.80)	80.69 (9.02)	80.35 (8.07)	74.82 (8.97)	72.66 (10.39)	73.06 (3.25)	75.38 (8.02)	88.20 ^{a} (9.54)
PC (O-34:1)	745	O-16:0/18:1	27.31 (7.01)	28.60 (5.03)	28.11 (3.96)	25.29 (0.92)	27.14 (6.94)	29.18 (9.39)	28.90 (7.98)	28.15 (1.97)	26.10 (2.72)	32.89^{a} (3.54)
PC (O-34:0)	747	O-18:0/16:0	4.81 (1.01)	4.96 (0.91)	5.89 (1.02)	5.89 (0.94)	5.79 (1.45)	5.27 (1.71)	4.87 (1.37)	4.42 (0.33)	4.87 (0.46)	5.76 (0.91)
PC (O-36:1)	773	O-18:0/18:1	14.31 (4.71)	13.61 (1.95)	14.90 (3.81)	12.70 (2.28)	13.93 (3.63)	14.03 (4.19)	15.08 (4.80)	12.79 (1.02)	12.37 (0.62)	15.38 ^{a} (2.73)
PC (O-36:0)	775	O-20:0/16:0	1.55 (0.31)	1.44 (0.13)	1.93 (0.38)	1.93 (0.13)	1.80 (0.42)	2.06 (0.77	1.96 (0.39)	1.58 (0.17)	1.69 (0.08)	2.43 ^{a} (0.60)
Total alkyl-acyl-PC			120.39 (21.70)	118.90 (18.43)	124.45 (14.41)	123.25 (12.19)	125.88 (19.47)	122.23 (23.79)	120.54 (23.92)	117.48 (5.31)	117.67 (9.77)	137.98^{a} (11.32)

Note: Data presented here as Mean ± SD of 4 mice in a group. Statistical analysis was done with a paired *t*-test, where $p < 0.05$ was considered as significant. Statistical significance is denoted as follows: ^{a} < 0.05, $^{b}p < 0.01$. WT vs. J20.

Table S3. Principal component loading (highest five species) of Pls-PE species at five different time points.

Timepoint	Pls-PE species	Loading	Raw *p* Value	Pls-PE species	Loading	Raw *p* Value
		PC1 (42.20%)			PC2 (26.20%)	
3 month	PE (P-18p1/22:2)	-0.22286	0.19	PE (P-16:0/24:6)	-0.28536	0.1
	PE (P-18:0/22:3)	-0.22113	0.16	PE (P-18:1/22:5)	-0.28178	0.15
	PE (P-18:0/22:4)	-0.21096	0.33	PE (P-18:0/22:6)	-0.25883	0.4
	PE (P-20:0/20:4)	-0.21057	0.25	PE (P-18:0/18:0)	-0.25218	0.08
	PE (P-18:1/22:4)	-0.20766	0.15	PE (P-18:0/18:2)	-0.24641	0.06
		PC1 (36.00%)			PC2 (31.60%)	
6 month	PE (P-18:0/20:4)	-0.22806	0.35	PE (P-16:0/22:3)	0.22327	0.11
	PE (P-16:0/22:4)	-0.22538	0.42	PE (P-18:1/20:2)	0.23779	0.06
	PE (P-20:1/20:4)	-0.21845	0.11	PE (P-18:0/22:4)	0.24735	0.02
	PE (P-16:0/20:3)	-0.21722	0.38	PE (P-18:1/22:3)	0.25948	0.002
	PE (P-18:1/22:6)	0.23442	0.03	PE (P-16:0/22:6)	-0.22207	0.07
9 month		PC1 (54.00%)			PC2 (23.00%)	
	PE (P-16:0/20:2)	-0.20595	0.03	PE (P-18:1/20:4)	-0.30747	0.05

	PE (P-16:0/22:3)	-0.20523	0.009	PE (P-16:0/22:5)	-0.30365	0.02
	PE (P-18:1/20:2)	-0.20515	0.01	PE (P-18:0/20:5)	-0.30223	0.01
	PE (P-18:0/18:2)	-0.20402	0.04	PE (P-16:0/20:4)	-0.23033	0.008
	PE (P-18:0/20:3)	-0.20361	0.02	PE (P-16:0/20:3)	-0.22394	0.007
12 month	PC1 (37.90%)			PC2 (29.40%)		
	PE (P-18:1/22:4)	0.22012	0.09	PE (P-18:0/18:1)	-0.25242	0.13
	PE (P-18:1/18:2)	0.22772	0.2	PE (P-18:1/18:0)	-0.25122	0.14
	PE (P-18:0/20:4)	0.22813	0.17	PE (P-18:0/18:0)	-0.24975	0.17
	PE (P-18:1/20:3)	0.22949	0.35	PE (P-16:0/20:1)	-0.24931	0.16
	PE (P-18:0/22:3)	0.23292	0.27	PE (P-16:0/20:0)	-0.23915	0.29
15 month	PC1 (47%)			PC2 (33.4%)		
	PE (P-16:0/24:4)	0.21278	0.001	PE (P-18:1/18:1)	0.25835	0.001
	PE (P-20:0/20:4)	0.21266	0.008	PE (P-18:0/22:3)	-0.25558	0.19
	PE (P-18:0/22:4)	0.21132	0.01	PE (P-16:0/20:0)	-0.25123	0.26
	PE (P-18:1/20:2)	0.21116	0.002	PE (P-18:1/18:0)	-0.24674	0.01
	PE (P-16:0/22:3)	0.20837	0.002	PE (P-18:0/20:5)	-0.24559	0.47

Note: Criteria: Autoscaling was used for data normalization. Loading value was selected irrespective of positive and negative signs.

Table S4. Principal component loading (highest five species) of Pls-PC species at five different time points.

Timepoint	Pls-PC Species	Loading	Raw *p* Value	Pls-PC species	Loading	Raw *p* Value
3 month	PC1 (40.60%)			PC2 (26.70%)		
	PC (P-16:0/22:6)	-0.33672	0.0001	PC (P-18:0/18:2)	-0.38945	0.22
	PC (P-16:0/16:1)	-0.33026	0.01	PC (P-18:0/18:1)	-0.38291	0.38
	PC (P-16:0/22:5)	-0.3151	0.07	PC (P-16:0/18:1)	-0.28331	0.27
	PC (P-18:0/16:0)	-0.30612	0.03	PC (P-18:0/22:6)	0.27982	0.0001
	PC (P-16:0/20:4)	-0.30477	0.0002	PC (P-16:0/16:0)	0.43365	0.23
6 month	PC1 (32.20%)			PC2 (29.70%)		
	PC (P-16:0/22:6)	-0.33672	0.07	PC (P-16:0/16:0)	0.35644	0.08
	PC (P-16:0/16:1)	-0.33026	0.29	PC (P-16:0/22:6)	0.40728	0.07
	PC (P-16:0/22:5)	-0.3151	0.47	PC (P-18:0/18:2)	-0.37247	0.03
	PC (P-18:0/16:0)	-0.30612	0.22	PC (P-18:0/20:2)	-0.36006	0.04
	PC (P-16:0/20:4)	-0.30477	0.007	PC (P-16:0/20:4)	-0.31506	0.007
9 month	PC1 (53.60%)			PC2 (18.10%)		
	PC (P-18:0/20:2)	-0.33161	0.27	PC (P-16:0/20:4)	-0.49645	0.47
	PC (P-16:0/22:5)	-0.33148	0.27	PC (P-20:0/16:0)	-0.28366	0.24
	PC (P-18:0/18:2)	-0.32327	0.34	PC (P-16:0/20:5)	0.34045	0.12
	PC (P-18:0/20:3)	-0.31354	0.32	PC (P-18:0/20:4)	0.36718	0.19
	PC (P-18:0/18:1)	-0.30923	0.42	PC (P-16:0/20:3)	0.46927	0.18
12 month	PC1 (32.40%)			PC2 (25.10%)		
	PC (P-18:0/16:0)	0.33347	0.43	PC (P-16:0/20:5)	-0.41387	0.22
	PC (P-16:0/22:5)	0.36207	0.35	PC (P-18:0/20:3)	-0.29676	0.07
	PC (P-18:0/18:1)	0.38914	0.12	PC (P-16:0/20:3)	0.4027	0.16
	PC (P-16:0/18:1)	0.39128	0.44	PC (P-16:0/20:4)	0.41338	0.11
	PC (P-18:0/18:2)	0.39792	0.13	PC (P-18:0/22:6)	0.44681	0.27
15 month	PC1 (53.60%)			PC2 (16.20%)		
	PC (P-18:0/20:2)	0.30127	0.42	PC (P-16:0/20:5)	-0.52488	0.43
	PC (P-16:0/22:5)	0.30894	0.09	PC (P-16:0/16:0)	-0.39196	0.12
	PC (P-18:0/22:6)	0.31313	0.03	PC (P-18:0/16:0)	-0.37846	0.31
	PC (P-18:0/18:2)	0.31362	0.32	PC (P-16:0/20:3)	-0.35975	0.009
	PC (P-16P:0/16:1)	0.3292	0.46	PC (P-18:0/20:4)	-0.24709	0.44

Note: Criteria: Autoscaling was used for data normalization. Loading value was selected irrespective of positive and negative signs.

Table S5. Principal component loading (highest five species) of alkyl-acyl-PC species at five different time points.

Time point	Alkyl-acyl-PC species	Loading	Raw p Value	Alkyl-acyl-PC species	Loading	Raw p Value
		PC1 (85.30%)			PC2 (6.90%)	
3 month	PC (O-16:0/18:1)	-0.47732	0.39	PC (O-20:0/16:0)	-0.82828	0.28
	PC (O-18:0/16:0)	-0.47345	0.41	PC (O-18:0/16:0)	0.036094	0.41
	PC (O-16:0/16:0)	-0.43783	0.39	PC (O-16:0/18:1)	0.097863	0.39
	PC (O-18:0/18:1)	-0.42904	0.39	PC (O-16:0/16:0)	0.11104	0.39
	PC (O-20:0/16:0)	-0.41503	0.28	PC (O-18:0/18:1)	0.53921	0.39
		PC1 (70.2%)			PC2 (16.5%)	
6 month	PC (O-18:0/16:0)	-0.49662	0.49	PC (O-16:0/16:0)	-0.69298	0.26
	PC (O-18:0/18:1)	-0.47624	0.18	PC (O-18:0/16:0)	0.43175	0.49
	PC (O-16:0/18:1)	-0.44389	0.11	PC (O-20:0/16:0)	-0.36762	0.49
	PC (O-20:0/16:0)	-0.40847	0.49	PC (O-18:0/18:1)	0.3998	0.18
	PC (O-16:0/16:0)	-0.40333	0.26	PC (O-16:0/18:1)	0.1959	0.11
		PC1 (89.80%)			PC2 (7.20%)	
9 month	PC (O-16:0/16:0)	0.43429	0.19	PC (O-20:0/16:0)	-0.72829	0.29
	PC (O-16:0/18:1)	0.46491	0.43	PC (O-16:0/18:1)	-0.23638	0.43
	PC (O-18:0/16:0)	0.44893	0.27	PC O-(18:0/18:1)	0.082755	0.48
	PC (O-18:0/18:1)	0.46525	0.48	PC (O-18:0/16:0)	0.29489	0.27
	PC (O-20:0/16:0)	0.42102	0.29	PC (O-16:0/16:0)	0.56561	0.19
		PC1 (89.3%)			PC2 (6.9%)	
12 month	PC (O-16:0/16:0)	0.43967	0.47	PC (O-16:0/16:0)	-0.60988	0.47
	PC (O-16:0/18:1)	0.45503	0.43	PC (O-16:0/18:1)	-0.42995	0.43
	PC (O-18:0/16:0)	0.45619	0.27	PC (O-18:0/16:0)	0.14211	0.27
	PC (O-18:0/18:1)	0.45192	0.19	PC (O-18:0/18:1)	0.37177	0.19
	PC (O-20:0/16:0)	0.43277	0.06	PC (O-20:0/16:0)	0.53365	0.06
		PC1 (70.70%)			PC2 (22.10%)	
15 month	PC (O-16:0/16:0)	-0.38223	0.04	PC (O-16:0/16:0)	-0.6282	0.04
	PC (O-16:0/18:1)	-0.46447	0.03	PC (O-18:0/16:0)	-0.52056	0.07
	PC (O-18:0/16:0)	-0.43092	0.07	PC (O-16:0/18:1)	0.23601	0.03
	PC (O-18:0/18:1)	-0.47744	0.04	PC (O-20:0/16:0)	0.3565	0.03
	PC (O-20:0/16:0)	-0.47384	0.03	PC (O-18:0/18:1)	0.38935	0.04

Note: Criteria: Autoscaling was used for data normalization. Loading value was selected irrespective of positive and negative signs.
